# An experimental study on cervix cancer with combination of HSV-TK/GCV suicide gene therapy system and ^60^Co radiotherapy

**DOI:** 10.1186/1471-2407-10-609

**Published:** 2010-11-06

**Authors:** Daozhen Chen, Qiusha Tang

**Affiliations:** 1 Clinical Laboratory, Wuxi Hospital for Matemaland Child Health Care Affiliated Nanjing Medical University, Wuxi, Jiangsu, 214002, China; 2 School of Clinical Medical Science, Southeast University, Nanjing 210009, China

## Abstract

**Background:**

To evaluate the killing effect of HSV-TK/GCV suicide gene therapy system combined with ^60^Co radiotherapy on human cervical cancer Hela cell line *in vitro *and *in vivo*, and to explore the radiosensitization by HSV-TK/GCV system.

**Methods:**

HSV-TK/GCV suicide gene therapy system and ^60^Co radiotherapy were used separately or in combination on human cervical cancer Hela cell line *in vitro *and *in vivo *to compare their effects. Colony formation test and the rate of radiosensitization effect (E/O) were employed to observed the radiosensitization by HSV-TK/GCV system.

**Results:**

HSV-TK/GCV suicide gene therapy system had strong therapeutic effects on Hela cells *in vitro *and *in vivo *(the inhibition rates were 45.8% and 39.5%, respectively), moreover, the combined administration of gene therapy and radiotherapy had stronger therapeutic effects *in vitro *and *in vivo *(the inhibition rate was 87.5% ***in vitro***, and the inhibition rate was 87.9% *in vivo*) (P < 0.01). The inhibition rate by radiotherapy alone was 42.4% ***in vitro ***and 35.8% *in vivo*. The sensitivity of combined therapy to radiotherapy increased more than that of therapy alone, the ability of colony formation decreased (P < 0.01). The rate of radiosensitivity effect (E/O) was 3.17(> 1.4), indicating HSV-TK/GCV system could exert a sensitizing effect on ^60^Co radiotherapy of the transplanted human cervical cancer cell in nude mice.

**Conclusion:**

HSV-TK/GCV system had radiosensitization. Gene therapy combined with radiotherapy may be a good supplementary method for cervix cancer synthetic treatment.

## Background

In recent years, molecular mechanism of tumor development and cell growth regulating mechanism are further studied, gene treatment of tumor has made rapid progress. More attention is paid to HSV-TK/GCV drug sensitive gene treatment system. But its clinical effect is not satisfactory, one of the reasons is that effective dose of GCV (100 mg/kg/1 d) may result in serious toxic side effect. Main complications are damage to renal function (40%~60%) and bone marrow inhibition (20%~41%) [1], which greatly influence and limit application scope and therapeutic effect of HSV-TK gene treatment. Radiotherapy is one of the most important clinical means in the treatment of tumor. Effect of radiotherapy depends on sensitivity of tumor cells and normal tissue to rays, but sensitivity is related to radiation dose. Excessive dose may damage normal tissue adjacent to tumor. So radiotherapy and gene treatment are both effective means but they have their own problems.

Tumor development is a complicated process involving many factors and gene incidents. Treatment of one link can not achieve satisfactory result. Some scholars suggested that combination of gene therapy, radiotherapy, chemotherapy and immunotherapy may enhance neoplastic suppressing effect. The suicide gene therapy and radiotherapy interact to produce a synergistic enhancement of cell kill may make the combined therapeutic effect even greater [2]. Atkinson et al [3] gave GCV and combined radiotherapy to mouse prostate cancer cells with transferred HSV-TK gene. They found that cell survival rate treated by simple HSV-TK/GCV system is 10%, 1% for simple radiotherapy group and 0.1% for combined treatment group. Its effect was not addition, but synergism. Pederson et al [4] used adenoviral as vector, transferred CD gene to cholecyst cancer cell system SK-chA-1 that was insensitive to radiation, added 5-FC for culture, combined with radiotherapy. Tumor cell death rate with simple 5-FC treatment was 20%~46%, it increased to 83.5%~91.5% with combined radiotherapy. Animal experiments also confirmed obvious growth inhibition of transplanted tumor in nude mice. Research of other scholars [5] also proved that effect of suicide gene system combined with radiotherapy was better than that of single treatment. Required titre of virus transfected vector, drug concentration of precursor and radiation dose were lower than those of single treatment. It could reduce adverse effect in treatment. So suicide gene system combined with radiotherapy may be a prospective scheme.

In present study, the author used reverse transcription virus to transfer HSV-TK gene into human cervical cancer Hela, combined with ^60^Co radiation to conduct *in vitro *and *in vivo *experiments, and explored its synergism and provided experimental and theoretical basis for clinical use.

## Methods

### Cell line and cell culture

Cervical cancer Hela cells were obtained from China Type Tissure Culture Collection Center (CCTCC, Wuhan, China); The Hela cells were maintained in RPMI1640 (Hangzhou Sijiqing Company, China) supplemented with 10% FBS in monolayers at 37°C and 5% CO_2_. All cells were passaged and harvested by standard trypsin (Quality Biologicals.Inc.)digestion at 37°C. Cells were routinely passaged at 80-90% confluence.

### vector

GINaTK retroviral vector was kindly provided by Dr. Daru Lu (Genetic Research Center of Fudan Univercity, Shanghai, China). The vector is based on the Moloney murine leukemia virus(MoMLV)long terminal repeat (LTR) and the cytomegalovirus (CMV) promoter. The vector included safety modifications to reduce the chances of retroviral vector recombination leading to the regeneration of replication-competent virus [6]. The HSV-TK gene was inserted downstream of the cytomegalovirus (CMV) promoter in a GINa plasmid containing NeoR selectable gene, the neomycin resistance gene encoding for NPT II(neomycin phosphotransferase II), which protects cells from the toxic effect of G418(a neomycin analog, Promega). NeoR has been used in many human clinical trials to date without adverse effect. Construction of package cell PA317/TK has been previously described [7].

### Retroviral transduction of Hela cell line

Supernatant from vector producer cells(**PA317/TK**) were obtained from cells that were 90-100% confluent. This supernatant was filtered through a 0.22 um filter, then replaced the cell growth medium of Hela by 5 ml viral supernatant, containing polybrene (8 μg/ml) to help the adhesion of viruses. Cells were returned to the incubator for 2 hours, then 5 ml of growth medium was added and incubated overnight. The next day the supernatant was removed and the cells were fed. After 2 days postinfection, we began the selection with 400 μg/ml of G418 (active drug, GIBCO) in RPMI1640. Cells were then observed daily and a large fraction of the cells died between days 4-and 7 of selection. Cells were kept under 800 μg/ml G418 selection for 14 days. The surviving HSV-TK-transduced cells(named Hela/TK) were pooled and used in the subsequent experiments.

### Experimental animals

BALB/C nude mice, female, 6-8 weeks old, body weight about 20 g, were from the Shanghai ExperimentalAnimal Center, the Chinese Academy of Sciences (Shanghai, China) [licence number:SCXK (Hu) 2002-0010]. All animal experiments were carried out in compliance with the national laws related to the conduct of animal experimentation.

### ^60^Co γ ray

^60^Co γ ray irradiator (Picker Zonegard V4 M60; Picker Interational, Cleveland, Ohio), absorptiondose rate was set at 168.5 cGy/min.

### *In vitro *experiment

#### Growth inhibition experiment of in *vitro *cells by HSV-TK gene combined with ^60^Co radiation

Hela/TK cells were inoculated in 96-well cell culture plate (10^3^cells/well), and cultured for 24 h, when cells grew on wall, they were treated as follows: ① gene treatment group: 10 μg/ml GCV solution was added to each well; ② ^60^Co radiotherapy group: each well was irradiated by ^60^Co γ ray, absorption dose was 400 cGy ③ combined treatment group: 10 μg/ml GCV was added to each well, and each well was radiated by ^60^Co γ ray, absorption dose was 400 cGy ④ control group: no treatment. Each experiment was performed in triplicate. Cells were cultured at 37°C in 5% CO_2 _incubator for 4 d, then 10 μl (5 mg/ml) MTT solution was added to each well of each group, then cultured for 4 h, finally 150 μl DMSO was added to each well, absorbance value A at 490 nm wavelength was determined, the well without cell was blank, Hela/TK without any treatment was the control, growth inhibition rate of cells was calculated with following formula. Inhibition rate (%) was (absorbance value A of control group minus absorbance value A of treatment group)/absorbance value A of control group × 100%

#### Flow Cytometry Assay

Hela/TK cells were inoculated in 6-well cell culture plate (5 × 10^3 ^cells/well), and cultured for 24 h, when cells grew on wall, they were treated as follows: ① gene treatment group: 10 μg/ml GCV solution was added to each well; ② ^60^Co radiotherapy group: each well was irradiated by ^60^Co γ ray, absorption dose was 400 cGy ③ Combned treatment group: 10 μg/ml GCV was added to each well, and each well was radiated by ^60^Co γ ray, absorption dose was 400 cGy ④ Control group: no treatment. Each experiment was performed in triplicate. Cells were cultured at 37°C in 5% CO_2 _incubator for 2 d. Then the cells of control group and experimental group were collected and rinsed in 0.1 PBS (pH 7.2 ~ 7.4) for three times, resuspended and fixed in 70% ethanol at 4 overnight. Cells were centrifuged, resuspended in 0.1 g/L RNase A at 37°C for 30 min and in 0.05 g/L propidium iodide at 4°C for 30 min. Cell cycle was analyzed by flow cytometer (FACS Vantage SE, Becton-Dickson Company).

#### Radiosensitization of HSV-TK/GCV tested by clone formation rate of cells

Hela/TK cells were passaged and harvested by standard trypsin (Quality Biologicals.Inc.)digestion, single cell was suspending in inoculum. Percentage of single cell was over 95%, 10^5 ^cells were inoculated in 6-well plate. Experiment was divided into 4 groups: ① control group; ② simple agent group (GCV content was 1/10 of effective concentration, i.e. l μg/ml); ③ simple radiation group, with certain dose of ^60^Co radiation, absorption dose was 400 cGy; ④ combined treatment group, first 1 μg/ml GCV (Cytovene-IV, Roche Laboratories, Nutley, NJ) was added and then radiated by ^60^Co, absorption dose was 400 cGy, which were cultured at 37°C in 5% CO_2 _incubator for 14 d, then each well was cleaned twice with PBS, dried in air, fixed by methanol for 15 min, stained with Giemsa for 15 min, and dye was washed away with running water, dried again in air, finally clone was counted more than 50 cells under microscope, per clone formation rate of cells was calculated by following formula: clone formation rate of cells (%) = (clone number/inoculated cell number) × 100%; relative clone formation rate of cells (%) = (clone formation rate of radiation group/clone formation rate of control group) × 100%

### *In vivo *experiment

#### Establishment of nude mice cervical cancer model and treatment of tumor

Forty BALB/c nude mice were divided into 4 groups: control group, gene treatment group, ^60^Co radiotherapy group and combined treatment group, 10 mice in each group. Each nude mouse was injected sc on both sides of flanks with 3.0 × 10^6 ^Hela/TK cells. When tumor grew to 1 cm in diameter, treatment as following: ① gene treatment group: GCV (100 mg/kg) was given intra-peritoneal cavity (i.p), injection volume 0.4 ml/mouse, once one day, continuous injection for 14 d; ② ^60^Co radiotherapy group: Synchronous with gene treatment group, tumor was radiated with ^60^Co once, absorption dose was 20 Gy; ③ combined treatment group: GCV (100 mg/kg) was given i.p, 2 h later, tumor tissue radiated with ^60^Co once, absorption dose was 20 Gy. Other steps were similar to gene treatment group: ④ Control group: same volume of aseptic phosphate buffer solution was given i.p, but without ^60^Co radiation. Four weeks later mice were sacrificed, tumor was resected and then their weight was weighed respectively with electro-balance.

#### Calculating average tumor inhibition rate of each group

Tumor inhibition rate (%) was calculated as (average tumor weight of control group minus average tumor weight of experimental group)/average tumor weight of control group × 100%.

#### Calculating radiosensitization

Radiosensitization was determined by E/O value (expected value/observed value) which was reported by Yunfei Xia et al [8] whether HSV-TK/GCV system had radiosensitization. When E/O > 1.4, it had radiosensitization. E = (T2/T1) × (T3/T1), which was expected value; 0 = (T4/T1), which was observed value. (T1, T2, T3 and T4 were respectively average tumor weights in control group, gene treatment group, ^60^Co radiotherapy group and combined treatment group).

#### Analytical and statistical methods

Experimental data were presented as means ± the standard deviations (SD) of four independent experiments performed in triplicate. Where appropriate, samples were analyzed by using the Student's two-tailed t-test or ANOVA(analysis of variance) by SPSS software. And statistical significance was defined as P < 0.05 and P < 0.01.

## Results

### Results of *in vitro *experiment

#### Cell growth inhibition

**Figure 1:**
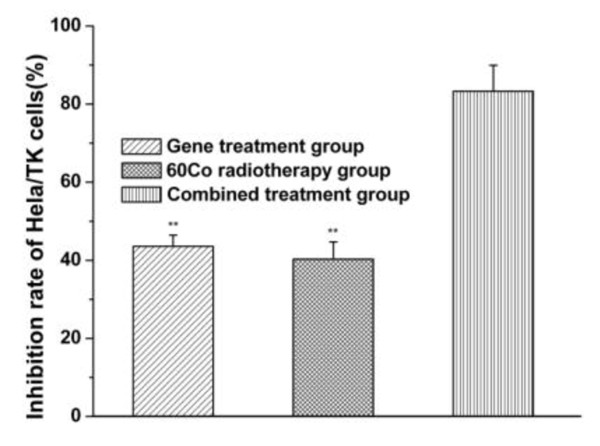
**MTT experiments showed the inhibition rates of Hela/TK cell growth induced by different methods and the inhibition rates were 45.8 ± 7.3%, 42.4 ± 5.6% and 87.5 ± 10.32% respectively**. Data were presented as the means ± SD of each experiments performed in triplicate. **P < 0.01 (gene treatment and ^60^Co radiotherapy group vs combined treatment groups) according to Student's t-test.

On 4^th ^day, inhibition rates of Hela/TK cell growth in the three treatment group were 45.8 ± 7.3%, 42.4 ± 5.6% and 87.5 ± 10.32% respectively, compared with gene treatment group and radiotherapy group, there was a significant difference in combined treatment group (*P *< 0.01) (See Figure 1).

#### Hela/TK cells apoptosis analyzed by FCMs

**Figure 2:**
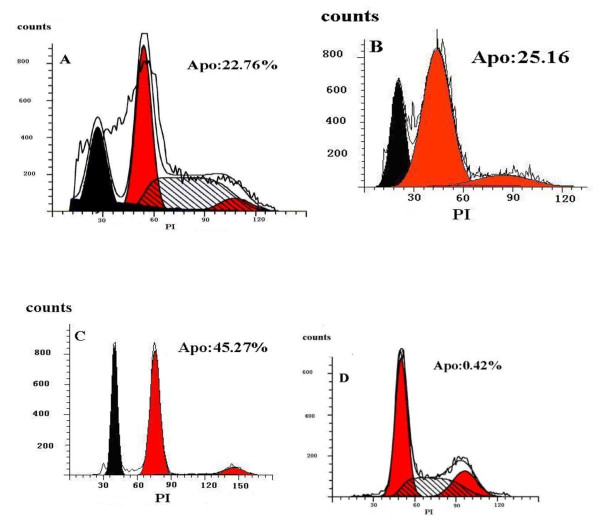
**Flow cytometry showed that apoptosis of Hela/TK cells was induced by the different methods**. A. gene treatment group; B. 60Co radiotherapy group; C. Combined treatment; D. control group, and the cells of apoptotic index (AI) were 22.76 ± 3.51%, 25.15 ± 3.17%, 45.27 ± 5.15, 0.42 ± 0.13% respectively.

The percentages of Hela/TK cells apoptosis were analyzed by FCMs on the fluorescence channel as described by Zhang [9]. The resulting histogram is a measure of PI staining, which is indicative of the amount of DNA in the nucleus to which the PI is bound. Hence, the histogram of PI staining is a reflection of chromatin content and the amount of apoptotic cells is in proportion to the sub-G0/G1-phase. According to the results shown in Figure 2, the percentages of apoptotic Hela/TK cells induced by Gene treatment, ^60^Co radiotherapy group and combined treatment were 22.76 ± 3.51%, 25.16 ± 3.17%and 45.27 ± 5.15%, respectively, as compared to 0.42 ± 0.13% apoptotic cells in the controls. Compared with gene treatment group and radiotherapy group, there was a significant difference in combined treatment group (*P *< 0.01).

#### Clone formation rate of cells in each group

There was no statistic significance in clone formation rate of cells of simple agent group (*P *> 0.05), compared with that of control group. whereas there was a significant difference between clone formation rate of simple radiotherapy group and clone formation rate of combined treatment group, compared with that of control group, their relative clone formation rates were 51% and 26% respectively (See table 1).

**Table 1:** Clone formation rates and relative clone formation rates of HeLa cells

	(%)	
**Group formation rate (%)**	**Clone formation rate (%)**	**relative clone**

Control(n = 3)	72 ± 11	-
Simple agent(n = 3)	69 ± 13	-
Simple radiotherapy (n = 3)	37 ± 6*	51 ± 11^#^
Combined treatment(n = 3)	19 ± 4*	26 ± 7

Note: Compared with control group, * P < 0.01; compared with combined treatment group, # P < 0.01

#### Results of *in vivo *experiment

After experiments, tumor mass of each group was weighed and tumor inhibition rates were calculated (see table 2). Results of *in vivo experiment *showed there were significant differences between the three treatment groups and control group, which indicated combined treatment group was superior to simple treatment group.

**Table 2:** Inhibition of transplanted subcutaneous tumor of mice (%)

Group	tumor weight (g)	tumor inhibition rate (%)
Control(n = 10)	2.22 ± 0.28	-
Radiotherapy(n = 10)	1.43 ± 0.05*	35.8 ± 4.9^▫^
Gene treatment(n = 10)	1.34 ± 0.14*	39.5 ± 4.0^▫^
Combined treatment(n = 10)	0.28 ± 0.04*	87.9 ± 2.1

Note: Compared with control group, * P < 0.01; compared with combined treatment group, ^▫ ^P < 0.01

#### Calculation of radiosensitization per tumor mass

E = (T_2_/T_1_) × (T_3_/T_1_) = 0.38; 0 = (T_4_/T_1_) = 0.12; E/0 = 3.17 (> 1.4). Above the results indicated the radiosensitization of HSV-TK/GCV suicide gene system was existing(E/O = 3.17 > 1.4).

## Discussion and conclusion

Tumor gene-radiotherapy is a new thought put forward in recent years to treat tumor. It transfers gene into the body, which can kill tumor and enhance sensitivity of radiation. It has synergism of radiation and gene in local radiotherapy [2]. Recent research has shown that HSV-TK can enhance sensitivity of radiation [10]. Many scholar hold that radiotherapy can enhance effect of suicide gene to inhibit tumor [11], and increase tumor cell apoptosis and tumor immunity with HSV-TK gene [12]. On the other hand, suicide gene therapy is one of the few gene treatment systems which may enhance radiation sensitivity [13]. Acyclic uzarin, propoxy uzarin and 5-FC have effect to enhance sensitivity. Kim et al [14] found that 9 L glioma transducted with HSV-TK gene was more sensitive to rays after GCV treatment twice 24 h before and after radiation. Sensitizer enhancement ratio (SER) is 1.6. Khil et al [15] transferred CD gene to intestine cancer cells, radiated for 72 h, and exposed them to 20 g/L 5-FC, then radiation sensitivity was increased 2.38 times compared with cells without transfected CD gene. In present study the author used HSV-TK/GCV suicide gene therapy system, combined with ^60^Co radiation, to conduct in vitro and in vivo experiments on human cervical cancer Hela. In in vitro the inhibition rates of Hela cell proliferation in the gene treatment group and radiotherapy group and combine treatment group were 45.8%, 42.4% and 87.5% respectively, compared with gene treatment group and radiotherapy group, difference in combined treatment group was significant (P < 0.01). The ratio of apoptosis among the gene treatment group, radiotherapy group and combined treatment group were (22.76 ± 3.51)%, (25.16 ± 3.17)%, (45.27 ± 5.15)% respectively compared with gene treatment group and radiotherapy group, difference in combined treatment group was significant (P < 0.01). Moreover, In vivo nude mice cervical cancer model treated experiment showed that there were significant differences in the three treatment groups, compared with control group; there was a significant difference in combined treatment group(P < 0.01), compared with gene treatment group or radiotherapy group, combined treatment group was superior to simple treatment group, which was in consistent with the study of NishiharaI et al [16]. In addition, the authors observed the radiosensitization of HSV-TK/GCV suicide gene system in the present study. Radiosensitization of HSV-TK/GCV was tested by clone formation rate of cells. The dosage of GCV was decided in 1/10 of effective concentration (i.e. l μg/ml) according to preliminary experiment result in order to remove proper cyto-toxic effect of GCV. The results indicated there was no statistic significance in clone formation rate of cells of simple agent group (P > 0.05), compared with that of control group, it meant that cyto-toxic effect of GCV was excluded. Whereas there was a significant difference between clone formation rate of simple radiotherapy group and clone formation rate of combined treatment group, compared with that of control group, their relative clone formation rates were 51% and 26% respectively (See table 1). It meant that combined treatment group was more sensitive to rays. Above the results indicated the radiosensitization of HSV-TK/GCV suicide gene system was existing(E/O = 3.17 > 1.4). Which was in consistent with the study of NishiharaI et al [16]. In short, in this present study in vitro and in vivo experiments showed HSV-TK/GCV system and ^60^Co may have synergism, when GCV less than effective dose was used in combined ^60^Co radiotherapy, sensitivity of HSV-TK gene to rays was enhanced significantly.

With regard to mechanism of the radiosensitization of HSV-TK/GCV suicide gene system, it was believed that when GCV enters cells transfected by HSV-TK gene, it converts into GCV-triphosphate, which can inhibit tumor cell repair of potentially lethal damage (PLD) and mix in DNA as competitive inhibitor of dTTP to enhance radiation sensitivity, reduce dose and toxic effect and improve therapeutic effect [17]. At present, the approach of HSV-TK/GCV suicide gene system combined radiotherapy on tumor therapy has not yet been used on clinical stage. But the investigation about wild type p53 gene inducing malignant cell apoptosis and its sensitization has been used on clinical stage. Swisher et al [18] had reported clinical stage II trial results about rAd2 p53 combined radiotherapy on nonsmall-cell lung cancer. The results showed: among the 19 patients, tumor cell has not yet been found by patho-biopsy in 63% (12/19). But tumor focus could be found in 16% (3/19) subjects accepting gene therapy, and the effective rate was less than 20% in subjects accepting radiotherapy. Those illustrated there were induction of p53 regulated genes and tumor regression in lung cancer patients after intratumoral delivery of adenoviral p53 (INGN 201) and radiation therapy.

On the whole, there were intimate relation between HSV-TK gene and tumor radiosensitivity. With molecule radiobiological development, it will be more clear that HSV-TK can enhance sensitivity of radiation and radiotherapy can enhance effect of suicide gene to inhibit tumor, which will give malignant tumor treatment more new approach and expectation; Meanwhile it will be very important to formulate correct combined therapeutic regimen and to predict curative effect and to appraise prognosis.

## Competing interests

The authors declare that they have no competing interests.

## Authors' contributions

QT conceived of the study, and participated in its design and coordination, and carried out in *vivo *animals experiments. DC carried out the *in vitro *experiments including growth inhibition experiment of cells and clone formation rate of cells experiment and apoptosis assay with flow cytometry, etc. and participated in the design of the study and performed the statistical analysis and drafted the manuscript. All authors read and approved the final manuscript.

## Pre-publication history

The pre-publication history for this paper can be accessed here:

http://www.biomedcentral.com/1471-2407/10/609/prepub
